# Deoxycholic Acid Impairs Human Sperm Quality and Function Through Oxidative Stress-Driven Damage

**DOI:** 10.3390/antiox14111271

**Published:** 2025-10-22

**Authors:** Steven Serafini, Elizabeth Pranov, Kaya Timova Bauer, Chika Onochie, Cristian O’Flaherty

**Affiliations:** 1 Department of Medicine, Experimental Medicine Division, McGill University, Montréal, QC H4A 3J1, Canada; 2 Department of Surgery, Urology Division, McGill University, Montréal, QC H4A 3J1, Canada; 3 The Research Institute, McGill University Health Centre, 1001 Decarie Blvd, Room EM02312, Montréal, QC H4A 3J1, Canada; 4 Department of Anatomy and Cell Biology, McGill University, Montréal, QC H3A 0C7, Canada; 5 Department of Pharmacology and Therapeutics, McGill University, Montréal, QC H3G 1Y6, Canada

**Keywords:** male infertility, obesity, sperm capacitation, oxidative stress, sperm function, deoxycholic acid (DCA), gut-testis axis

## Abstract

Infertility is a growing global health concern, with male infertility contributing to nearly half of all cases. While conventional semen analysis often overlooks functional impairments, oxidative stress has emerged as a key factor affecting sperm quality. Notably, oxidative stress is elevated in obesity, a rising epidemic affecting more than 1 in 8 people worldwide. This study examines the role of deoxycholic acid (DCA), a secondary bile acid that is elevated in obesity, and its potential to induce oxidative stress and impair sperm function. Semen samples from healthy donors were incubated with DCA, and its effects on sperm motility, viability, capacitation, and oxidative stress markers were assessed. Sperm motility and viability were evaluated using computer-assisted semen analysis (CASA) and hypo-osmotic swelling (HOS) tests, while sperm capacitation was measured via tyrosine phosphorylation (P-Tyr) and acrosome reaction (AR). Oxidative stress markers were quantified using flow cytometry. While progressive motility and viability remained unchanged, DCA reduced hyperactive motility, P-Tyr, and acrosome reaction and increased oxidative stress markers in spermatozoa. These findings suggest that secondary bile acids can disrupt sperm function through oxidative mechanisms, affecting non-conventional semen parameters that may go undetected in standard analyses. This underscores the gut-testis axis’s role in male infertility and highlights the need for more comprehensive diagnostics and targeted therapies.

## 1. Introduction

Human infertility, a disease of the male or female reproductive system defined by the failure to achieve a pregnancy after 12 months or more of regular unprotected sexual intercourse, is increasingly recognized as a pressing global public health challenge, affecting approximately one in six couples worldwide [1,2,3]. It is a multifactorial condition influenced by a complex interplay of genetic predispositions, development, environmental exposures, and modifiable lifestyle factors [4]. Male infertility accounts for nearly 50% of all infertility cases, with an estimated 34% remaining idiopathic despite extensive advances in reproductive medicine [5]. Compounding this issue, a sustained global decline in semen quality has been observed over recent decades, raising concerns about broader population-level reproductive health trends [6]. Concurrently, the obesity epidemic has emerged as a critical determinant of reproductive dysfunction [7]. More than one-third of the global population is classified as obese, with men comprising 43% of this group [8]. Alarmingly, the global prevalence of obesity impacts not only the adult population but also children and adolescents. This trend is particularly concerning, as early-life obesity has been linked to disturbances in the normal development of the male reproductive system, potentially resulting in long-term impairments in fertility [9]. In general, excess adiposity adversely affects male fertility through mechanisms involving hormonal dysregulation [10,11], altered testicular function [12,13,14], and chronic inflammation and oxidative stress [15,16,17,18,19,20].

Among the mechanisms contributing to male infertility, oxidative stress has emerged as a central factor, arising from an imbalance between reactive oxygen and nitrogen species (RONS) production and the body’s antioxidant defences, leading to molecular and cellular damage. Mammalian spermatozoa are particularly susceptible due to their limited intrinsic antioxidant capacity. Unlike somatic cells, sperm lack cytoplasmic organelles such as peroxisomes, key sites for ROS detoxification, as they contain catalase, as these structures are eliminated during spermiogenesis [21]. Consequently, sperm are highly prone to oxidative damage, which can impair fertilization potential and adversely affect early embryonic development.

Sperm capacitation is a highly orchestrated and time-sensitive sequence of biochemical and biophysical transformations required including production of RONS, activation of adenylyl cyclase and phosphorylation signalling pathways driven by protein kinase A, C, ERK, PI3K/AKT and protein tyrosine kinases, increase in intracellular pH and calcium, among others, for the acquisition of hyperactivated motility and enabling the spermatozoon to recognize and bind to the zona pellucida, initiate the AR, and ultimately fertilize the oocyte [22,23,24,25]. Low physiological levels of ROS are essential for sperm capacitation and AR [26,27]; whereas elevated ROS induce lipid peroxidation [28], DNA fragmentation [29], and mitochondrial dysfunction [30], collectively compromising sperm quality and reproductive outcomes. Both excessive ROS production and diminished antioxidant defence can disrupt sperm function. Key ROS, including hydrogen peroxide (H_2_O_2_), superoxide anion (O_2_^•−^), and nitric oxide (NO), can profoundly impair sperm function [31].

Recent evidence has identified gut microbiota dysbiosis, an imbalance in the composition and function of intestinal microbial communities, as a critical factor associated with obesity and having far-reaching systemic effects [32]. The gut microbiome plays a central role in regulating host metabolism, including energy homeostasis and fat storage [33], and its disruption has been linked to a spectrum of metabolic disorders and chronic systemic inflammation [34]. Of relevance to reproductive health is the emerging concept of the gut-testis axis, which postulates a relationship between gut microbial composition and male reproductive function. Dysbiosis within this axis has been correlated with male subfertility phenotypes such as oligozoospermia (low sperm count) [35] and teratozoospermia (abnormal sperm morphology) [36]. A key microbial metabolite implicated in this context is deoxycholic acid (DCA), a secondary bile acid derived from cholic acid through microbial metabolism in the gut [37]. Diets rich in saturated fats, commonly linked to obesity, have been shown to elevate intestinal DCA concentrations [38], and growing evidence suggests that DCA promotes oxidative stress by enhancing ROS production [39].

Given the essential role of oxidative balance in regulating sperm physiology, elevated ROS levels induced by DCA may impair key functional processes such as sperm capacitation. Disruption of redox homeostasis during capacitation could therefore compromise sperm function and reduce fertilization potential. Despite growing insights, the precise molecular mechanisms linking gut microbiota dysbiosis to altered sperm function remain incompletely understood and warrant further investigation.

This study tests the hypothesis that elevated levels of DCA impair sperm capacitation-associated modifications, including tyrosine phosphorylation (P-Tyr), hyperactive motility, and AR through the induction of oxidative stress, thereby compromising fertilization potential without altering standard semen parameters. By uncovering the role of microbial metabolites in male reproductive health, this work aims to identify novel targets for infertility treatment and promote more balanced, male-inclusive approaches to reproductive care.

## 2. Materials and Methods

### 2.1. Materials

Deoxycholic Acid (DCA) (264101), mouse monoclonal anti-P-Tyrosine (P-Tyr), clone 4G10 (#05-321), nitrocellulose blotting membrane (GE10600004), Progesterone (P0130), Pisum sativum lectin conjugated with FITC (PSA-FITC) (L0770) purchased from Millipore Sigma Canada (Oakville, ON, Canada). Horseradish peroxidase-conjugated goat anti-mouse IgG (#115-035-062) and donkey anti-rabbit IgG (#711-035-152) antibodies were purchased from Jackson Laboratories (Bar Harbor, ME, USA). Percoll (#45-001-747), enhanced chemiluminescence (ECL) Western blotting Substrate (PI32106), SYTOX™ Blue (S34857), Mitochondrial Superoxide Indicator (MitoSOX™) (M36008), JC-1 (T3168), and BODIPY™ 581/591 C11 (D3861) were all sourced from ThermoFisher Scientific (Markham, ON, Canada). Anti-8-hydroxy-2′-deoxyguanosine (ab183393) antibody was purchased from Abcam (Toronto, ON, Canada). All other chemicals used were of reagent grade and purchased from Sigma-Aldrich (Milwaukee, WI, USA). DY 268 was purchased from Tocris (Burlington, ON, Canada). Human fetal cord serum samples were collected from the Cellular Therapy Laboratory at the Research Institute, McGill University Health Centre. Fetal cord ultrafiltrates (FCSu) were prepared using Amicon Ultra-4 filter devices (UFC8010D) with membranes having a 3 kDa exclusion limit (MilliporeSigma, Oakville, ON, Canada), following previously established procedures [40].

### 2.2. Subjects and Sperm Sample Preparation

Ethical approval for this study was granted by the McGill University Health Centre Research Ethics Board, and written informed consent was obtained from all 15 participants. To reduce the potential for donor-related bias, each experiment was conducted using semen samples from different individuals. Samples were obtained from healthy male donors aged 18–30 years following 72 h of sexual abstinence. After collecting, the semen was incubated at 37 °C for 30 min to allow for liquefaction. Sperm cells were then isolated using a discontinuous four-layer Percoll gradient (20%, 40%, 65%, and 95%), with highly motile sperm recovering from the 95% layer and the interface between the 65% and 95% layers. This method enriches motile sperm while minimizing the presence of abnormal spermatozoa, round cells, and other contaminants. Sperm motility was quantitatively evaluated using the Hamilton Thorne computer-assisted sperm analysis (CASA) system with HTCASAII software version 1.17 (Beverly, MA, USA). Only samples exhibiting progressive motility greater than 70% were used. Sperm concentration was determined using an Improved Neubauer hemacytometer and adjusted to 50 × 10^6^ spermatozoa/mL with Biggers, Whitten, and Whittingham (BWW) medium, which contains 91.5 mM NaCl, 4.6 mM KCl, 1.7 mM CaCl_2_, 1.2 mM KH_2_PO_4_, 1.2 mM MgSO_4_, 5.6 mM D-glucose, 0.25 mM sodium pyruvate, 21.6 mM sodium lactate, and 20 mM HEPES at pH 7.95 [41]. The sperm samples were then incubated at 37 °C for 3.5 h with or without 10% *v*/*v* fetal cord serum ultrafiltrate (FCSu), an established inducer of human sperm capacitation [27,42], or various concentrations (0–100 µM) of DCA [43,44]. FCSu induces similar capacitation-related changes as BSA/bicarbonate (e.g., increased protein tyrosine phosphorylation, hyperactivation, and responsiveness to AR inducers). Because BSA interferes with reactive oxygen species (ROS) measurements [40], we omitted BSA as an inducer of capacitation in our experiments. Sperm capacitation was assessed by evaluating spermatozoa’s P-Tyr levels and spermatozoa’s ability to undergo progesterone-induced AR, well-established markers of human sperm capacitation [45]. We then determined the impact of DCA treatments on sperm viability/motility and oxidative stress markers, as described below.

### 2.3. Sperm Viability and Motility Determinations

Sperm viability was evaluated using a modified version of the hypo-osmotic swelling (HOS) test [46,47]. Post-treatment, sperm samples were gently combined with 150 μL of hypo-osmotic solution containing 1.5 mM fructose and 1.5 mM sodium citrate, followed by incubation at 37 °C for 30 min. The treated samples were then mounted onto Superfrost Plus microscope slides for analysis. Viability assessment was performed using a Leica DFC 450C microscope at 200× magnification, supported by Leica Application Suite X (LASX) software (version 1.1.0.12420; Leica Microsystems, Wetzlar, Germany). For each sample, two independent counts of 200 sperm cells were conducted. Only spermatozoa displaying characteristic tail swelling or the presence of a cytoplasmic droplet, indicative of intact membrane function, were classified as viable.

Sperm motility was assessed using a HT-IVOS II computer-assisted sperm analysis (CASA) system (Hamilton Thorne, Beverly, MA, USA) [48,49]. Each sample was supplemented with bovine serum albumin (BSA) at a final concentration of 3 mg/mL, thoroughly mixed, and subsequently transferred to a pre-warmed at 37 °C Makler chamber and maintained at 37 °C during the determination. A minimum of 200 spermatozoa per sample was evaluated to determine total, progressive, and hyperactivated motility. By convention, all spermatozoa entering the field in the first 10 frames are counted during acquisition. Note that any sperm departing the field in the first 10 frames are not counted to determine concentration and motility correctly. Sperm motility parameters were defined as per described in the WHO manual [46]. Total motility was expressed as the proportion of motile sperm relative to the overall sperm count. Progressive motility was defined as the percentage of sperm with a time-averaged path velocity (VAP) of ≥25 µm/s and a straightness (STR) of ≥80%. Hyperactivation was characterized by a curvilinear velocity (VCL) ≥ 150 µm/s, linearity (LIN) ≤ 50%, and an amplitude of lateral head displacement (ALH) ≥ 7.0 µm [46,50].

### 2.4. Acrosome Reaction (AR) Determination

Following an incubation for 3.5 h at 37 °C with or without FCSu and DCA, sperm samples were washed twice by centrifugation at 600× *g* for 5 min to remove any proteins shed during sperm capacitation. The pellets were resuspended in BWW containing 10 µM progesterone, as described by Baldi et al. [51], and incubated for 30 min at 37 °C. After a second centrifugation at 600× *g* for 5 min, the supernatant was discarded, and the pellets were resuspended in HOS solution for 30 min at 37 °C to assess cell viability. Later, they were centrifuged at 600× *g* for 5 min and resuspended in 95% ethanol. A 10 µL aliquot of ethanol-fixed sperm was placed onto Superfrost slides. Without allowing the sample to dry, 20 µL of PSA-FITC (30 µg/mL) was added, and the slides were incubated at 37 °C for 5 min. After incubation, the slides were rinsed with distilled water, dried, and a drop of prolonged antifade-DAPI solution was applied. The slides were then covered with coverslip. To assess acrosomal integrity, the percentage of spermatozoa with intact acrosomes (fluorescence present in the acrosome) and reacted acrosomes (absence of fluorescence) was determined by analyzing 200 spermatozoa per sample. Imaging was performed using a Zeiss LSM780 Laser Scanning Confocal Microscope (Opti-Tech Scientific, Montréal, QC, Canada) at 100× magnification.

### 2.5. SDS-PAGE and Immunoblotting

After 3.5 h of incubation, sperm protein samples were mixed with a sample buffer containing 100 mM dithiothreitol and a phosphatase inhibitor cocktail, then heated at 100 °C for 5 min. Following this, the samples were centrifuged at 21,000× *g* for 5 min at room temperature. The supernatant was loaded onto a 10% polyacrylamide gel, and electrophoresis was performed at a constant current of 0.025 amps per gel. After electrophoresis, proteins were electrotransferred to nitrocellulose membranes for 45 min at 100 volts. The membranes were then blocked with 5% skim milk in Tris-buffered saline containing 0.1% Tween 20 (TTBS) for 1 h. Incubation with the primary antibody, anti-P-Tyr (1:10,000 dilution), was carried out at room temperature for 1 h. Following washes with TTBS, the membranes were incubated with the appropriate horseradish peroxidase-conjugated secondary antibodies (goat anti-mouse or donkey anti-rabbit) for 45 min at room temperature. Protein bands were visualized using chemiluminescence on an Amersham Imager 680 (GE Healthcare, Montréal, QC, Canada).

After immunoblotting, the nitrocellulose membranes were then stained using a silver stain solution (2% *w*/*v* trisodium citrate, 0.8% *w*/*v* FeSO_4_, and 0.2% *w*/*v* AgNO_3_ in deionized water). Loading controls were also confirmed by incubating the same membrane with anti-α-Tubulin (1:10,000) antibody. The membrane was then imaged using the Amersham Imager 680.

The relative intensities of the protein bands (e.g., 105 kDa and 85 kDa for P-Tyr) were quantified using FIJI ImageJ software (version 2.1.0/1.53c, National Institutes of Health, Stapleton, NY, USA), with normalization to the loading control bands (105 kDa and 65 kDa) from the silver stain or α-Tubulin. For further analysis, the relative intensity of each sample’s protein bands was normalized to the corresponding values from the non-capacitated control sample. The protein band intensities were expressed as mean ± standard error.

### 2.6. Sperm DNA Oxidation

DNA oxidation in sperm treated with DCA was evaluated by measuring the level of 8-hydroxy-2-deoxyguanosine (8-OHdG) in cells exposed to FCSu 10% *v*/*v* and varying concentrations of DCA (50 or 100 µM) for 3.5 h at 37 °C, in accordance with a previously established protocol [49]. Following treatment, spermatozoa were washed and incubated with 2 mM dithiothreitol (DTT) in phosphate-buffered saline (PBS) for 45 min at 37 °C to promote chromatin decondensation. After washing, cells were incubated with an anti-8OHdG FITC-conjugated antibody (1:1000 dilution) in 0.1% Triton X-100 and 0.1% sodium citrate for 30 min at 37 °C, protected from light. The samples were then washed and stained with Sytox Blue^TM^ to quantify the percentage of FITC-positive cells relative to Sytox-stained cells. Flow cytometry was performed using the MACSQuant Analyzer with a 488 nm argon laser.

### 2.7. Mitochondrial Membrane Potential

Mitochondrial membrane potential (MMP) was assessed according to a previously established protocol [49,52] using JC-1 (a cationic carbocyanine dye). Sperm samples were treated with or without FCSu 10% *v*/*v* and DCA (50 or 100 µM) for 3.5 h at 37 °C. The samples were incubated with 2 μM JC-1 for 15 min at 37 °C in the dark. Following incubation, cells were washed and then incubated with 0.2 μM Sytox Blue^TM^ to exclude dead cells during flow cytometry analysis. At high MMP, JC-1 forms J-aggregates in the mitochondria, which emit red fluorescence detected in the B2 channel of the 488 nm argon laser. In a low MMP state, JC-1 remains in its monomeric form, emitting green fluorescence on the B1 channel. The results were expressed as the ratio of red to green fluorescence intensity, with a decrease in this ratio indicating mitochondrial depolarization.

### 2.8. Lipid Peroxidation Determination

Lipid peroxidation was determined using a BODIPY 581/591 C11 probe, that was used in the past to demonstrate levels of lipid peroxidation in spermatozoa [53,54], as done before [49,55,56]. Briefly, spermatozoa were treated with or without FCSu 10% *v*/*v* and DCA (50 or 100 µM) for 3.5 h at 37 °C. Samples were stained with 5 μM BODIPY 581/591 C11 for 30 min at 37 °C in the dark. A positive control was prepared under the same conditions with 40 μM ferrous sulphate (FeSO_4_). Afterward, the cells were washed and stained with 0.2 μM Sytox Blue^TM^ to exclude dead cells during analysis. A minimum of 10,000 events was analyzed for each sample using a MACSQuant Analyzer flow cytometer. Data is presented as the percentages of live spermatozoa having a positive BODIPY C11 signal (green fluorescence) [57].

### 2.9. Determination of Mitochondrial Superoxide Production

Mitochondrial O_2_^•−^ production was assessed in spermatozoa by flow cytometry using MitoSOX, a lipid-soluble cation that selectively targets mitochondria and is oxidized by O_2_^•−^ and fluoresces red upon binding to nucleic acid [58]. Briefly, spermatozoa were incubated with or without FCSu 10% *v*/*v* and DCA (50 or 100 µM) for 3.5 h at 37 °C. After incubation, samples were stained with 2 μM of MitoSOX and 0.2 μM of Sytox Blue^TM^ for 15 min at 37 °C. Then, the spermatozoa were washed and resuspended in HBS, and they were analyzed by flow cytometry. The positive control for MitoSOX labelling was prepared by incubating a sperm aliquot with 40 μM of Antimycin A for 3.5 h at 37 °C. Data were analyzed as a percentage of cells producing mitochondrial O_2_^•−^.

### 2.10. Statistical Analysis

All data are expressed as mean ± standard error of the mean (SEM). Statistical comparisons between groups were performed using one- or two-way ANOVA followed by Tukey’s, Bonferroni’s, or Šídák’s multiple comparisons post hoc tests, as appropriate, using GraphPad Prism version 10 (GraphPad Software, Inc., San Diego, CA, USA). Assumptions of normality and homogeneity of variances were verified using the Shapiro-Wilk test and Levene’s test, respectively. Differences were considered statistically significant at *p* ≤ 0.05.

## 3. Results

### 3.1. Deoxycholic Acid Impairs Sperm Capacitation

**Figure 1:**
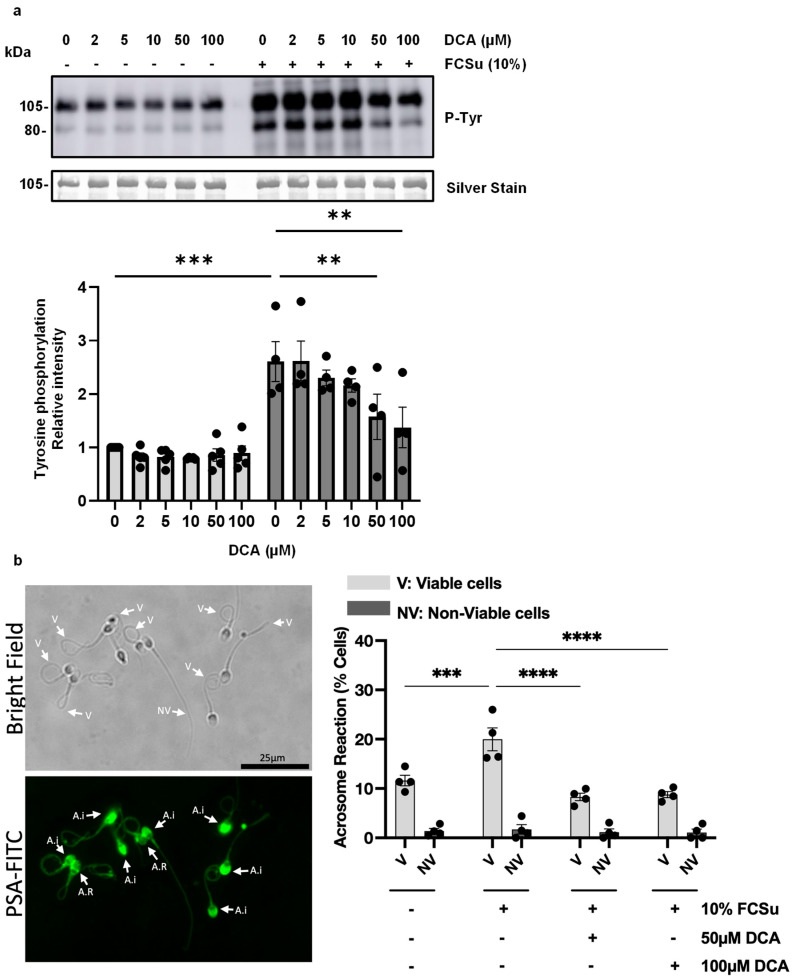
**Effects of DCA on sperm capacitation**. (**a**) Phospho-tyrosine (P-Tyr) intensity of the 80- and 105 kDa protein bands in spermatozoa either untreated (-) or treated (+) with Fetal Cord Serum Ultrafiltrate (FCSu, 10% *v*/*v*) and varying concentrations of deoxycholic acid (DCA, 50 or 100 μM) for 3.5 h at 37 °C. Both 50 and 100 μM DCA mitigated the FCSu-induced increase in P-Tyr. Signal quantification was normalized to each lane’s silver-stain optical density. (**b**) Progesterone-induced acrosome reaction (AR) in spermatozoa pre-treated with or without FCSu (10% *v*/*v*) and DCA (50 or 100 μM). Following pre-treatment, sperm samples were incubated in hyperosmotic solution (HOS) for 30 min at 37 °C to assess viability, then exposed to 10 μM progesterone to induce AR. The AR was measured as the percentage of live spermatozoa with non-intact acrosomes using PSA (Pisum sativum agglutinin) staining and fluorescence microscopy. The data represent sperm samples from four healthy donors (n = 4). A.I.: acrosome intact; A.R.: acrosome reacted. Statistical analysis was performed using one- or two-way ANOVA followed by Tukey’s or Bonferroni’s post hoc tests. Significant differences are indicated as ** *p* ≤ 0.01, *** *p* ≤ 0.001 and **** *p* ≤ 0.0001.

In our study, P-Tyr levels of prominent bands 105 kDa and 80 kDa (a well-established marker of sperm capacitation) were assessed in spermatozoa treated with or without FCSu 10% *v*/*v* and varying doses of DCA (0, 2, 5, 10, 50, 100 µM). In non-treated spermatozoa, DCA did not have an impact on P-Tyr levels (Figure 1a). FCSu-treated spermatozoa with concentrations of 50 and 100 µM of DCA demonstrated a significant dose-dependent decrease in P-Tyr (Figure 1a). Moreover, FCSu-treated spermatozoa with 50 and 100 µM of DCA had a significant reduction in progesterone-induced AR (a hallmark of sperm capacitation) in live spermatozoa (Figure 1b). In addition, FCSu alone did not promote AR in non-treated spermatozoa. These results demonstrate that FCSu-mediated capacitation is disrupted by DCA treatment. Further experimentation was performed using 50 and 100 µM of DCA.

### 3.2. DCA Treatment Does Not Affect Sperm Total and Progressive Motility and Viability, Yet Impairs Hyperactive Motility

**Figure 2:**
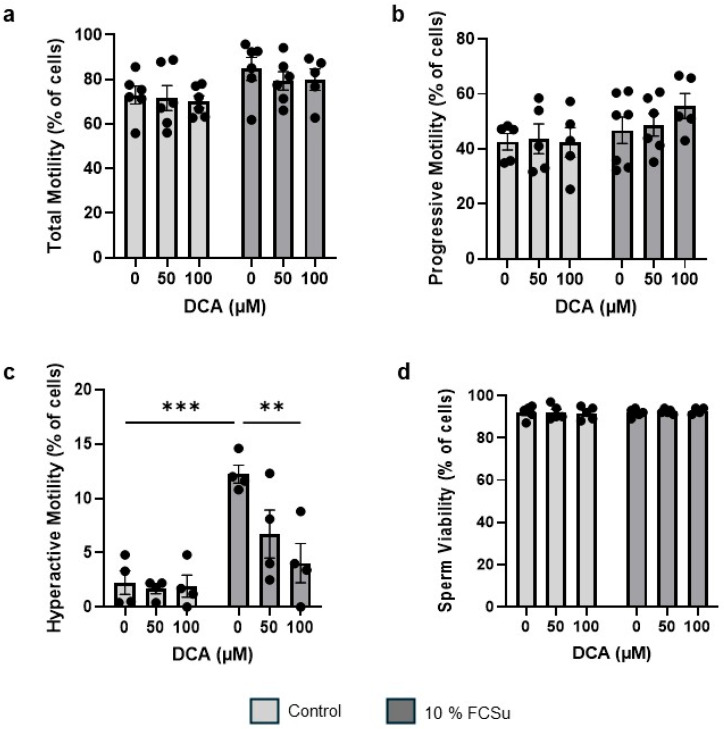
**Effects of DCA on sperm motility and viability**. Spermatozoa were incubated with or without FCSu (10% v/v) and DCA (50 or 100 μM) for 3.5 h at 37 °C. (**a**,**b**) Treatment with DCA at 50 or 100 μM did not impair (**a**) total motility or (**b**) progressive motility in either the control or FCSu groups. (**c**) DCA at 50 or 100 μM reduced hyperactive motility in both control and FCSu groups. (**d**) DCA treatment did not affect sperm viability as assessed by the hyperosmotic swelling (HOS) test. Data represent sperm samples from four to seven healthy donors (n = 4–7). Statistical analysis was performed using two-way ANOVA followed by Tukey’s test. Significant differences are indicated as ** *p* ≤ 0.01, and *** *p* ≤ 0.001.

To assess the impact of increasing concentration of DCA on sperm motility and viability, we incubated spermatozoa without (non-capacitated) or with FCSu (capacitating spermatozoa) with 50 and 100 µM of DCA. Both total (Figure 2a) and progressive (Figure 2b) motility remained unchanged across all DCA concentrations tested. As expected, hyperactive motility (a distinctive motility characterized by non-linear sperm movement with a high amplitude flagellar beating that is observed in capacitated spermatozoa) significantly increased in FCSu-treated spermatozoa compared to non-capacitated controls (Figure 2c), indicated by the orange tracks (Supplementary Figure S2a). Interestingly, a dose-dependent reduction in hyperactive motility was observed at 50 µM and 100 µM of DCA in FCSu-treated sperm (Figure 2c), resulting in a statistically significant decrease with 100 µM of DCA (*p* ≤ 0.01). Sperm viability, as determined by the HOS test, confirmed that DCA exposure did not affect sperm viability (Figure 2d). Hence, DCA exposure strictly impairs human sperm functional capacity rather than viability.

### 3.3. DCA Exposure Increases Oxidative Stress in Human Spermatozoa

**Figure 3:**
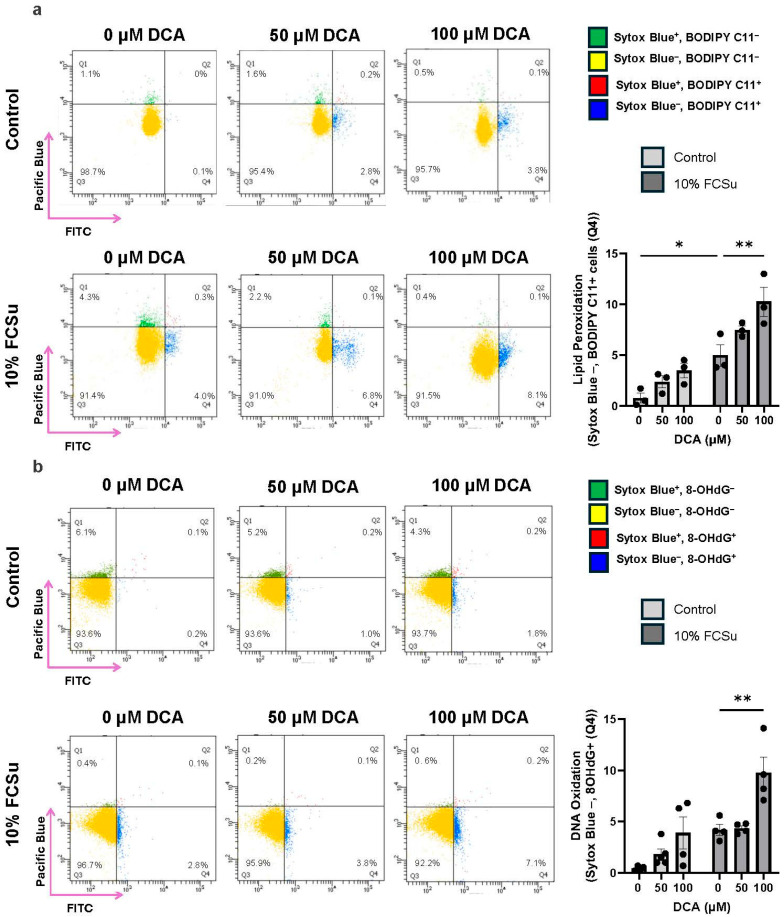
**Effects of DCA on lipid peroxidation and DNA oxidation in human spermatozoa**. Spermatozoa were incubated for 3.5 h at 37 °C with or without (control) fetal cord serum ultrafiltrate (FCSu, 10% *v*/*v*) and DCA (50 or 100 μM). After washing, samples were incubated with the following probes: (**a**) BODIPY-C11 (5 μM) for lipid peroxidation together with Sytox Blue (0.2 μM) for viability; and (**b**) anti-8-OHdG FITC-conjugated antibody (1:1000) for DNA oxidation together with Sytox Blue (0.2 μM) for viability. Flow cytometry was used to identify live (Q3 and Q4) and dead (Q1 and Q2) spermatozoa, distinguishing (**a**) BODIPY-C11^−^ (Q3) and BODIPY-C11^+^ (Q4), or (**b**) 8-OHdG^−^ (Q3) and 8-OHdG^+^ (Q4) populations. Data are presented as the percentage of live spermatozoa positive for BODIPY-C11 or 8-OHdG. Results represent sperm samples from healthy donors (n = 4). Statistical analysis was performed using two-way ANOVA followed by Tukey’s test. Significant differences are indicated as * *p* ≤ 0.05 and ** *p* ≤ 0.01.

We observed that spermatozoa capacitated with FCSu in the presence of 100 µM DCA exhibited a significant increase in the percentage of live cells displaying markers of oxidative stress, compared to untreated controls and cells exposed to either FCSu alone or lower concentrations of DCA. Specifically, lipid peroxidation was assessed using BODIPY-C11, a fluorescent probe that detects oxidized membrane lipids. A marked increase in BODIPY-C11 fluorescence was observed in the 100 µM DCA group, indicating elevated lipid peroxidation levels (Figure 3a). In parallel, DNA oxidative damage was quantified using 8-hydroxy-2′-deoxyguanosine (8-OHdG). Spermatozoa treated with 100 µM DCA showed a significantly higher proportion of 8-OHdG-positive cells, highlighting increased DNA oxidation under these conditions (Figure 3b). These findings suggest that high-dose DCA promote oxidative damage in capacitating spermatozoa.

**Figure 4:**
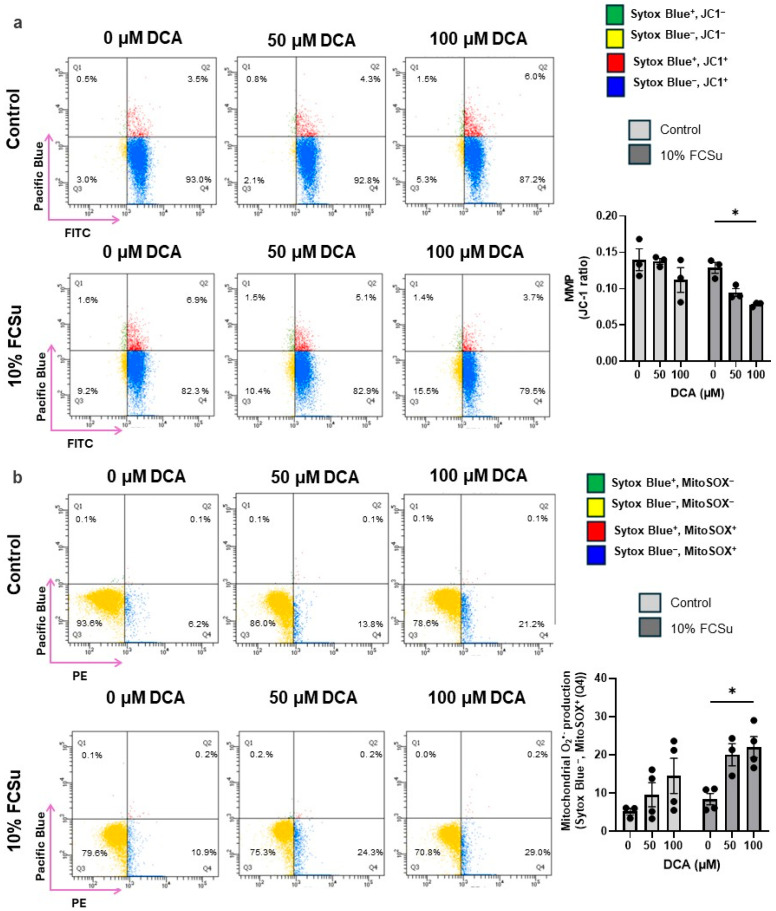
**Effects of DCA on mitochondrial O_2_^•−^ production and membrane depolarization in human spermatozoa.** Sperm samples were incubated with or without (control) fetal cord serum ultrafiltrate (FCSu, 10% *v*/*v*) and DCA (50 or 100 μM) for 3.5 h at 37 °C. After washing, samples were incubated with the following probes: (**a**) JC-1 (2 μM) for mitochondrial membrane potential and Sytox Blue (0.2 μM) and (**b**) MitoSOX (2 μM) for mitochondrial superoxide (O_2_^•−^) production and Sytox Blue (0.2 μM). Flow cytometry was used to identify live (Q3 and Q4) and dead (Q1 and Q2) spermatozoa, distinguishing MitoSOX^−^ (Q3) and MitoSOX^+^ (Q4), or (**b**) the ratio of red/green fluorescence intensity indicating depolarization when the ratio decreases. Data are presented as the percentage of live spermatozoa positive for MitoSOX. Results represent sperm samples from healthy donors (n = 4). Statistical analysis was performed using two-way ANOVA followed by Tukey’s test. Significant differences are indicated as * *p* ≤ 0.05.

Moreover, we further investigated the impact of DCA treatment on mitochondrial membrane potential (MMP) and mitochondrial O_2_^•−^ production in spermatozoa, key indicators of mitochondrial dysfunction and oxidative stress. A significant depolarization of the mitochondrial membrane was observed in the 100 µM DCA-treated spermatozoa, as evidenced by reduced JC-1 aggregate formation, suggesting compromised mitochondrial integrity (Figure 4a). Concurrently, these spermatozoa exhibited increased intracellular mitochondrial O_2_^•−^ production (Figure 4b).

Altogether, these results demonstrate that exposure to high concentrations of DCA during sperm capacitation not only enhances lipid and DNA oxidation but also disrupts mitochondrial function and generation of mitochondrial O_2_^•−^, compromising sperm quality and function.

### 3.4. DCA Does Not Exert Its Effects via the Farnesoid X Receptor (FXR)

**Figure 5:**
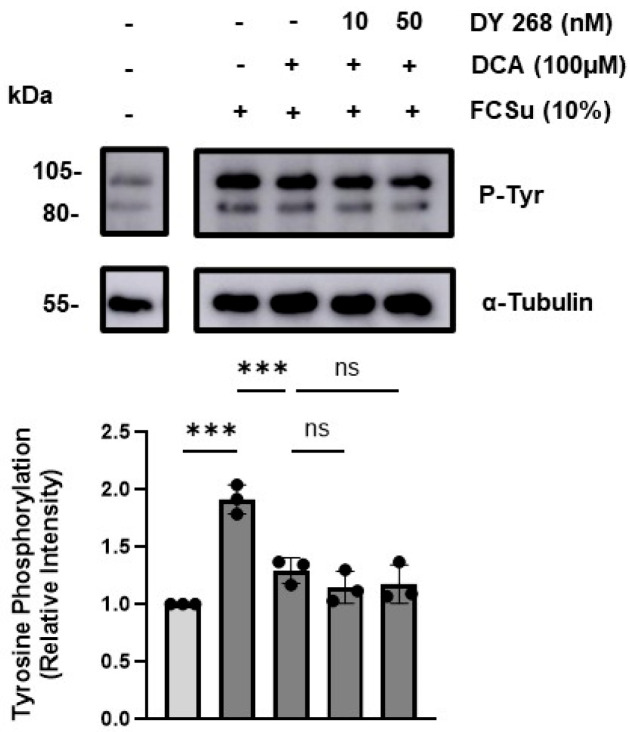
Effects of farnesoid X receptor (FXR) inhibitor on DCA-mediated inhibition of sperm capacitation. Phospho-tyrosine (P-Tyr) intensity of the 80 and 105 kDa protein bands in spermatozoa either untreated (-) or treated (+) with Fetal Cord Serum Ultrafiltrate (FCSu, 10% *v*/*v*), deoxycholic acid (DCA) at 100 μM, and FXR inhibitor (DY 268) at 10 or 50 nM for 3.5 h at 37 °C. 100 μM of DCA mitigated the FCSu-induced increase in P-Tyr, but DY 268 at 10 or 50 nM did not restore FCSu-induced P-Tyr. Signal quantification was normalized to each lane’s α-tubulin optical density. Bands shown belong to the same blot. Results represent sperm samples from three different healthy donors (n = 3). Statistical analysis was performed using one-way ANOVA with Šídák’s multiple comparisons test. Significant differences are indicated as *** *p* ≤ 0.001. N.S.: not significant differences.

To investigate whether DCA mediates oxidative damage and functional impairment in spermatozoa through FXR, a receptor of bile acids. We inhibited FXR using the potent antagonist DY268 at two concentrations (IC_50_, 10 nM, and 50 nM). Inhibition of FXR did not prevent the decrease in P-Tyr levels following treatment with 100 µM DCA (Figure 5), indicating that DCA’s effects are independent of FXR signalling.

## 4. Discussion

This study provides novel insights into how gut microbiota-derived deoxycholic acid (DCA) impairs sperm capacitation through oxidative stress, without altering conventional semen parameters. Our findings demonstrate that DCA exposure significantly increases oxidative damage in capacitating spermatozoa, manifested as lipid peroxidation, DNA oxidation, and mitochondrial dysregulation, while sparing sperm motility and viability. This decoupling between standard semen quality metrics and functional impairment underscores a critical limitation in routine fertility diagnostics and suggests a potential mechanism for idiopathic male infertility.

Our findings provide new insights into how DCA perturbs the delicate redox regulation required for successful human sperm capacitation. Sperm capacitation is a tightly regulated maturation process enabling spermatozoa to fertilize the oocyte. It is intrinsically associated with a physiological increase in RONS production that functions as a signalling cue [23,59]. While physiological RONS levels during capacitation are required for signalling, DCA exposure shifted this redox balance toward a pathological state, exacerbating oxidative damage. Oxidative stress is a well-established contributor to male infertility in 30–80% of cases [26,60,61], with ROS capable of inducing lipid peroxidation, protein oxidation, and DNA damage in spermatozoa. However, standard semen analysis, which primarily evaluates motility, morphology, and viability, may overlook functional impairments caused by sub-lethal oxidative stress. In our study, DCA exposure significantly increased lipid peroxidation (as measured by C11-BODIPY) (Figure 3a) and DNA oxidation (Figure 3b) (as indicated by 8-OHdG), and mitochondrial dysfunction (Figure 4a,b) without affecting overall motility or viability (Figure 2). These findings suggest that ROS levels induced by DCA are insufficient to compromise sperm survival but high enough to disrupt key molecular processes, including those required for successful fertilization. The observed decline in P-Tyr (Figure 1a), AR (Figure 1b) and hyperactivation (Figure 2c), hallmarks of sperm capacitation, further illustrate a functional impairment that would not be detected in standard male fertility testing.

Mechanistically, DCA’s capacity to disrupt sperm function likely begins with its interaction with the plasma membrane. As an amphipathic secondary bile acid, DCA can integrate into lipid bilayers and alter membrane fluidity, permeability, and protein conformation [62,63]. In somatic cells, bile acids are internalized via both passive diffusion and carrier-mediated uptake through transporters such as the apical sodium-dependent bile acid transporter (ASBT/SLC10A2) and organic anion transporting polypeptides (OATP/SLCO) superfamily [64]. OATP isoforms (e.g., Oatp1a5, 3a1,6b1,6c1 and 6d1) are present in spermatogonia [65], potentially enabling DCA uptake by the human spermatozoa. Once internalized, DCA localizes to mitochondrial membranes, where it perturbs their structural order and can trigger mitochondrial dysfunction and oxidative stress [66].

Alternatively, DCA may exert its effects by interacting with specific plasma membrane receptors. To explore this, we assessed the role of FXR in mediating DCA-induced oxidative stress and the impairment of human sperm functional capacity, given its reported localization to the midpiece of human spermatozoa [67]. Our results indicate that FXR does not mediate DCA-induced impairment of capacitation (Figure 5). Moreover, in skeletal muscle fibres, DCA has been shown to decrease mitochondrial membrane potential, reduce expression of oxidative phosphorylation complexes, and increase mitochondrial ROS production, partially via a plasma membrane receptor TGR5 [68]. TGR5, although it has not been localized in human spermatozoa, according to the Human Proteome Atlas (https://www.proteinatlas.org, accessed on the 8 August 2025), is found in male reproductive tissue like the prostate, seminal vesicles and the epididymis, but not in the testis. Thus, it is probable that the TGR5 receptor can be loaded into spermatozoa during epididymal sperm maturation and respond to the elevated levels of DCA prompted by gut dysbiosis. However, so far, there are no studies indicating that TGR5 is part of the cargo of epididymosomes and further studies are necessary to test this hypothesis.

In human spermatozoa, whose energy metabolism relies heavily on OXPHOS in the midpiece, DCA-driven disruptions can rapidly elevate mitochondrial O_2_^•−^ levels, overwhelming antioxidant defences. This mechanism is consistent with our observations of increased mitochondrial O_2_^•−^ (Figure 4a), decreased mitochondrial membrane potential (Figure 4b), and impaired sperm capacitation (Figure 1a,b). Future studies should aim to localize the TGR5 receptor in human spermatozoa and determine whether it contributes to capacitation impairment by mediating oxidative stress through targeted interventions.

**Figure 6:**
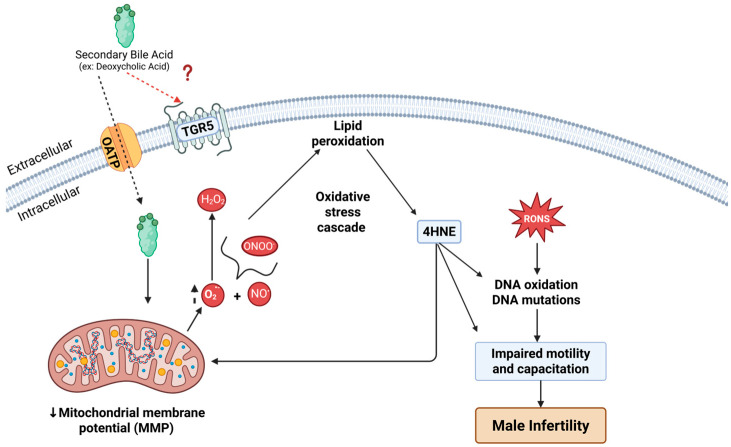
**Deoxycholic acid (DCA) impairs sperm capacitation by promoting oxidative stress.** DCA enters spermatozoa via unidentified organic anion transporting polypeptides (OATP) and may also activate the plasma membrane bile acid receptor TGR5 (possible involvement indicated by the red question mark). Within mitochondria, DCA perturbs membrane dynamics, causing depolarization and disruption of oxidative phosphorylation, which elevates the production of reactive oxygen species (ROS; O_2_^•−^, H_2_O_2_) and reactive nitrogen species (RNS; NO, ONOO^−^). Lipid peroxidation generates 4-hydroxynonenal (4-HNE), which further amplifies reactive oxygen and nitrogen species (RONS) and drives oxidative damage to proteins, lipids, and DNA. This oxidative cascade impairs key sperm functions, including capacitation, hyperactivated motility, and the AR, ultimately contributing to male infertility. Created in BioRender. Lab, O. (2025) https://BioRender.com/qkrkg88 (accessed on 16 August 2025).

Notably, the high abundance of polyunsaturated fatty acids (PUFAs) in the sperm plasma membrane renders these cells particularly vulnerable to oxidative stress. Lipid peroxidation involves a cascade of complex reactions that oxidize PUFAs, producing lipid hydroperoxides (ROOH) [69]. ROOH, being relatively unstable, is further degraded into cytotoxic secondary products such as aldehydes, ketones, carboxylic acids, and 4-hydroxynonenal (4-HNE) [70,71]. The accumulation of lipid peroxidation products, notably 4-HNE, under DCA exposure is particularly concerning. These highly reactive electrophiles can form covalent adducts with proteins, including those in the electron transport chain [72] such as succinate dehydrogenase, thereby creating a self-perpetuating cycle of ROS generation and lipid peroxidation (Figure 6) [73,74].

In terms of genomic integrity, 4-HNE can also react with DNA to form adducts that promote mutations and affect the paternal genome [49,52,75]. Furthermore, ROS can directly oxidize DNA bases, particularly guanosine, generating 8-OHdG [76], a well-established biomarker of sperm DNA oxidative damage [49,52,58]. Such alterations have been linked to reduced fertilization potential, impaired embryo development, and adverse pregnancy outcomes [77,78,79].

Limitations of our work include that in vitro capacitation in BWW with FCSu cannot fully replicate the complex biochemical environment of the female reproductive tract, which provides antioxidant defences and signalling factors that may modulate DCA effects. Additionally, while the DCA concentrations tested are physiologically relevant in the context of obesity and gut dysbiosis, in vivo exposure dynamics remain to be determined.

The key clinical implication of this study is that oxidative stress-mediated sperm dysfunction may occur in men with normal semen analysis results, potentially contributing to unexplained infertility. Given the link between gut dysbiosis, DCA, and oxidative stress-dependent damage, functional sperm assays that measure oxidative stress and the ability to undergo capacitation should be considered as adjuncts to standard semen analysis. Furthermore, the observation that DCA impairs sperm function without affecting overall motility suggests that oxidative damage could also undermine assisted reproductive technology outcomes, even when spermatozoa appear morphologically normal and motile. Incorporating oxidative stress screening into sperm selection protocols for IVF and ICSI may help identify sublethal defects and improve reproductive success rates.

## 5. Conclusions

Our findings establish a mechanistic link between gut microbiota-derived metabolites and male reproductive dysfunction, highlighting oxidative stress as a hidden mediator of sperm capacitation failure. By revealing that DCA impairs functional parameters without altering conventional semen metrics, this work underscores the need to broaden diagnostic approaches for male infertility. It points to the gut-testis axis as a potential therapeutic target.

## Data Availability

Data are contained within the article.

## Supplementary Materials

The following supporting information can be downloaded at: https://www.mdpi.com/article/10.3390/antiox14111271/s1, Figure S1: Entire blots corresponding to Figure 1; Figure S2: CASA sperm motility tracks; Figure S3: Entire blots corresponding to Figure 5.

## Abbreviations

The following abbreviations are used in this manuscript:

8-OHdG	8-hydroxy-2′-deoxyguanosine
4-HNE	4-hydroxynonenal
ALH	Amplitude of lateral head displacement
AR	Acrosome reaction
ASBT	Apical sodium-dependent bile acid transporter
BSA	Bovine serum albumin
BWW	Biggers, Whitten, and Whittingham medium
CASA	Computer-assisted semen analysis
DAPI	4′,6-diamidino-2-phenylindole
DCA	Deoxycholic acid
DNA	Deoxyribonucleic acid
DTT	Dithiothreitol
ECL	Enhanced chemiluminescence
FCSu	Fetal cord serum ultrafiltrate
FITC	Fluorescein isothiocyanate
FXR	Farnesoid X receptor
HBS	HEPES-buffered saline
HEPES	4-(2-hydroxyethyl)-1-piperazineethanesulfonic acid
HOS	Hypo-osmotic swelling test
IC_50_	Half maximal inhibitory concentration
IgG	Immunoglobulin G
IVF	In vitro fertilization
JC-1	5,5′,6,6′-Tetrachloro-1,1′,3,3′-tetraethylbenzimidazolylcarbocyanine iodide
LIN	Linearity
MMP	Mitochondrial membrane potential
NO	Nitric oxide radical
O_2_^•−^	Superoxide anion
OATP	Organic anion transporting polypeptides
OXPHOS	Oxidative phosphorylation
PBS	Phosphate-buffered saline
PSA	Pisum sativum agglutinin
PUFA	Polyunsaturated fatty acids
P-Tyr	Phospho-tyrosine
RNS	Reactive nitrogen species
ROOH	Lipid hydroperoxides
ROS	Reactive oxygen species
RONS	Reactive oxygen and nitrogen species
SEM	Standard error of the mean
STR	Straightness
TGR5	G-protein coupled bile acid receptor 1 (Takeda G-protein receptor 5)
TTBS	Tris-buffered saline with Tween 20
VAP	Average path velocity
VCL	Curvilinear velocity
WHO	World Health Organization
