# Supplementary material for: Deoxycholic Acid Impairs Human Sperm Quality and Function Through Oxidative Stress-Driven Damage
Source: Antioxidants (Basel). 2025 Oct 22;14(11):1271. doi: 10.3390/antiox14111271 (PMC12649610; doi:10.3390/antiox14111271)

## Supplementary Figure. 1 – Entire blots corresponding to Figure 1

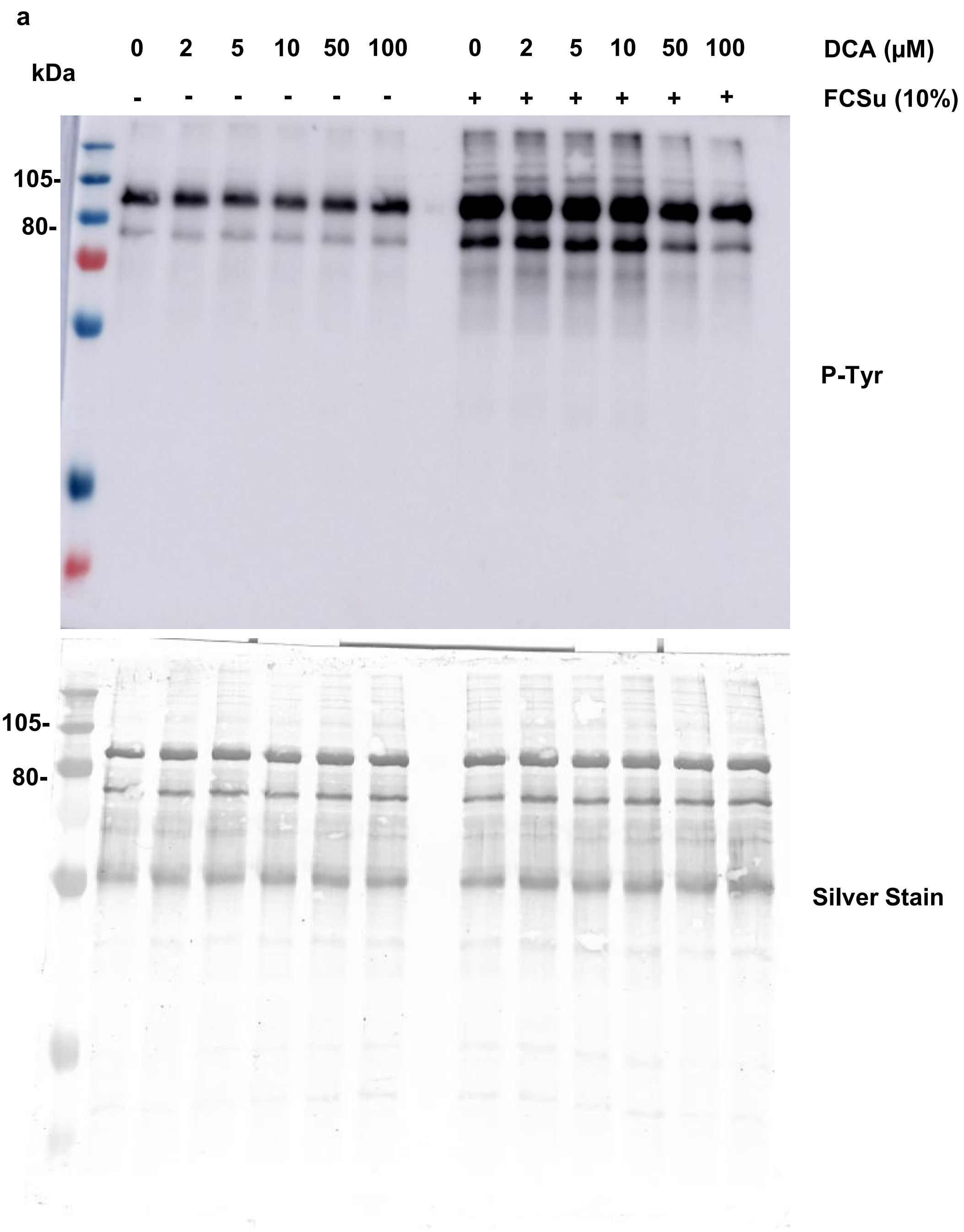

Supplemental Figure S2- CASA Sperm motiltiy tracks

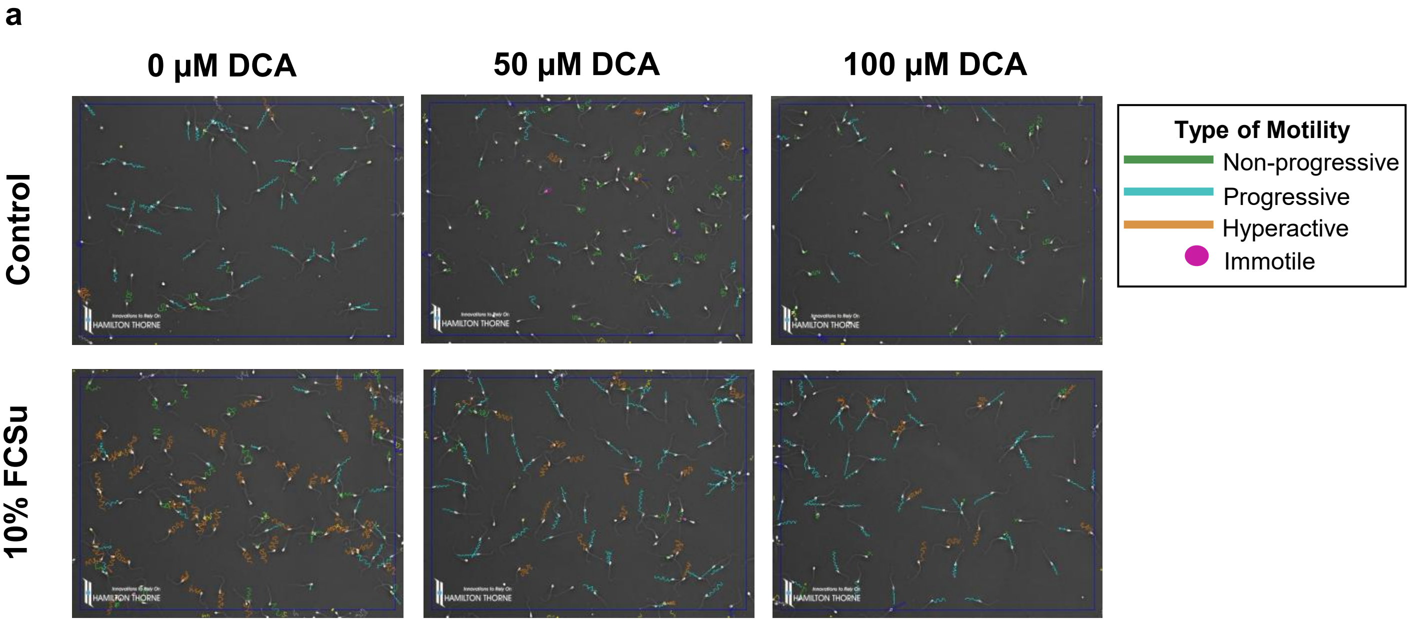

**Supplemental Figure 3 – Entire blots corresponding figure 5**

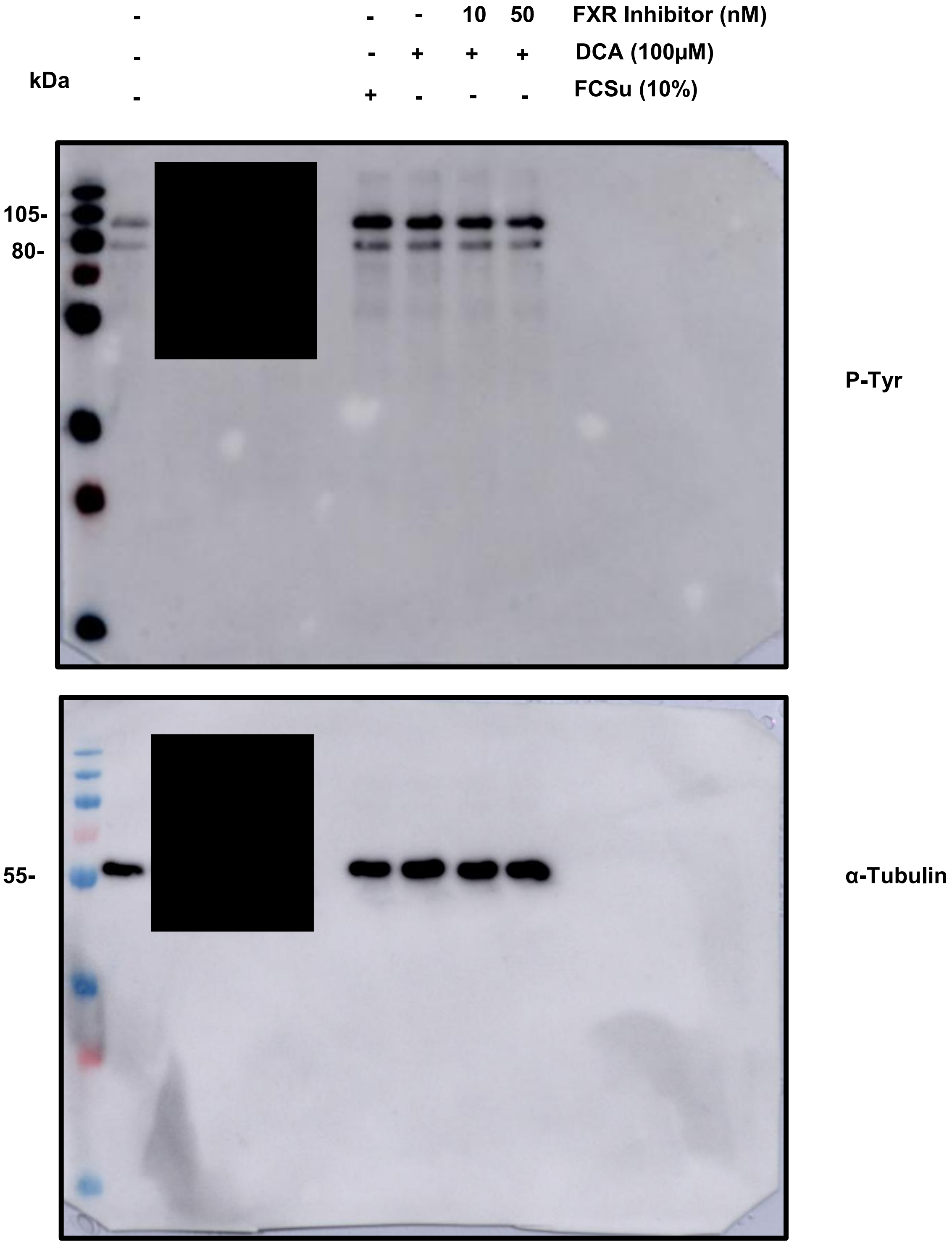

Supplement: Supplementary file 1 [file antioxidants-14-01271-s001.zip › antioxidants-3867970-supplementary.pdf]
